# Partially supervised exercise programmes for fall prevention improve physical performance of older people at risk of falling: a three-armed multi-centre randomised controlled trial

**DOI:** 10.1186/s12877-024-04927-0

**Published:** 2024-04-03

**Authors:** Anne-Gabrielle Mittaz Hager, Nicolas Mathieu, Sophie Carrard, Alice Bridel, Christina Wapp, Roger Hilfiker

**Affiliations:** 1 School of Health Sciences, HES-SO Valais-Wallis, University of Applied Sciences and Arts Western Switzerland, Valais, Sion Switzerland; 2 Bern University of Applied Sciences, Department of Health Professions, Bern, Switzerland; 3 ARTORG Center for Biomedical Engineering Research, University of Bern, Bern, Switzerland; 4 School of Health Sciences, HES-SO Valais-Wallis, Rathaustrasse 25, 3941 Leukerbad, Switzerland

**Keywords:** Older people, Home-based exercise programme, Falls, Functional mobility, Balance

## Abstract

**Background:**

Falls have a major impact on individual patients, their relatives, the healthcare system and related costs. Physical exercise programmes that include multiple categories of exercise effectively reduce the rate of falls and risk of falling among older adults.

**Methods:**

This 12-month, assessor-blinded, three-armed multicentre randomised clinical trial was conducted in adults aged ≥ 65 years identified as at risk of falling. Four hundred and five participants were randomly allocated into 3 groups: experimental group (*n* = 166) with the Test&Exercise partially supervised programme based on empowerment delivered with a tablet, illustrated manual and cards, reference group (*n* = 158) with the Otago partially supervised programme prescribed by a physiotherapist delivered with an illustrated manual and control group (*n* = 81) with the Helsana self-administrated programme delivered with cards. Experimental and reference groups received partially supervised programmes with 8 home sessions over 6 months. Control group received a self-administered program with a unique home session. The 3 groups were requested to train independently 3 times a week for 12 months. Primary outcome was the incidence rate ratio of self-reported falls over 12 months. Secondary outcomes were fear of falling, basic functional mobility and balance, quality of life, and exercise adherence.

**Results:**

A total of 141 falls occurred in the experimental group, 199 in the reference group, and 42 in the control group. Incidence rate ratios were 0.74 (95% CI 0.49 to 1.12) for the experimental group and 0.43 (95% CI 0.25 to 0.75) for the control group compared with the reference group. The Short Physical Performance Battery scores improved significantly in the experimental group (95% CI 0.05 to 0.86; *P* = 0.027) and in the reference group (95% CI 0.06 to 0.86; *P* = 0.024) compared with the control group.

**Conclusion:**

The self-administered home-based exercise programme showed the lowest fall incidence rate, but also the highest dropout rate of participants at high risk of falling. Both partially supervised programmes resulted in statistically significant improvements in physical performance compared with the self-administered programme.

**Trial registration:**

NCT02926105. ClinicalTrials.gov. Date of registration: 06/10/2016.

**Supplementary Information:**

The online version contains supplementary material available at 10.1186/s12877-024-04927-0.

## Introduction

Between 28 and 35% of community-dwelling older adults over 65 years of age fall each year and the frequency of falls increases with age and frailty level [1]. Falls have a major impact on individual patients, their relatives, the healthcare system and related costs [2].

Physical exercise programmes that include multiple categories of exercise effectively reduce the rate of falls and risk of falling among older adults when delivered in group classes or individually at home [3], except for older people following hospital discharge [4]. To maintain the effects of such exercise programmes, older people should continue the training for as long as possible [5].

Different types of exercise programme can be offered to older people to engage them in continuing exercise, namely supervised, partially supervised, or self-administered programmes. Home-based exercise programmes often require less resources than supervised exercise programmes [6], however participation and adherence to home-based exercise programmes generally remains low [7].

To sustain exercise adherence we developed an innovative partially supervised home-based exercise programme, named Test-and-Exercise, based on the concepts of self-efficacy, self-confidence and empowerment, delivered via an application on an Android tablet [8].

The primary aim of this study was to compare the effectiveness on fall prevention in older people of partially supervised versus self-administered home-based exercise programmes. Secondary objectives were to compare their effects on physical abilities, quality of life and exercise adherence.

## Method

### Design

This study was a three-arm parallel-group, assessor-blinded, multicentre randomised controlled trial. The study protocol has been published [8]. Recruitment occurred over a period of 4 years, from 3 August 2016 to 23 November 2020. Participants were recruited in partner institutions (hospitals, healthcare centres, and medical offices) by doctors, physiotherapists and nurses. They informed potential participants about the study and asked them whether they agreed to be contacted by the local coordinator. In case of agreement, care providers completed a pre-recruitment scale and sent it to the coordinator. The local coordinators met the potential participants at their homes to explain the study protocol in detail and to verify that they met the eligibility criteria. Participants were given a consent form and a stamped addressed envelope to send their consent if they agreed to participate. Participants were then invited to take part in the initial assessment (t_0_), carried out by blinded physiotherapist assessors, before the randomisation.

An independent researcher created a computer-generated random sequence allocation list stratified for urban or rural settings, age groups (65–79; ≥ 80 years) and categories of risk of falling (low-moderate; high) based on the STEADI algorithm [9] with random block sizes, using the command "ralloc" within Stata. The list was integrated into the software for Research Electronic Data Capture and was concealed from members of the research team [10]. Couples living in the same house were allocated to the same group. The project assistant manually triggered randomisation after the baseline assessment. Local coordinators noted the allocation group, using this software.

The intervention took place over a period of 6 months. Participants allocated in both partially supervised programmes received eight one-to-one physical therapy sessions and four phone calls at 6-week intervals. At each session the physical therapist revised the completion of the exercise, verified that the fall and training diaries were correctly completed, and repeated the recommendations for the training. Participants allocated in the self-administered home-based programme received a single one-to-one physical therapy session and four phone calls at 6-week intervals. All participants of the three groups were asked to train three times a week for 30–45 min, with a rest day between training days. Participants were encouraged to walk on the days between training days. At each phone call, the physical therapists asked about exercises and encouraged participants to train themselves and reminded them to complete their diaries.

After the intervention period, the participants were invited for an intermediary assessment (t_1_). To ensure that the assessors remained unaware of the group allocation, participants were instructed not to discuss their intervention with the assessor. The participants then continued to practise the exercise programme allocated to them independently for a further 6 months (follow-up). After 12 months, they were invited for a final assessment (t_2_).

This study is reported according the recommendations of the extension of the CONSORT 2010 Statement for multi-arm parallel-group randomised trials [11].

### Participants, therapists, centres

Eligible participants were aged ≥ 65 years, living independently at home, able to walk without mobility aids in their home, with a history of falls in the previous 12 months or of perceiving fear of falling (score ≥ 20 on the Fall Efficacy Scale-International [12]), and good understanding of French or German. Participants were excluded in case of: severe vision impairments that did not permit reading the booklet/tablet or completing the monthly diaries; undergoing physical therapy that included balance training; having cognitive impairments assessed with a score < 24 points on the Mini-Mental State Examination scale [13]; or if participation was contraindicated by the treating physician.

All the physiotherapists were specially trained for the interventions for which they were responsible (assessment or home treatment). This study was conducted in the Lausanne area, in the French- and German-speaking parts of Valais (Switzerland).

### Interventions

#### Experimental programme

The Test-and-Exercise programme is an individualised, partially supervised, home-based balance and strength training programme delivered by a trained physical therapist. The development of this programme is described in the protocol [8]. It contains 50 physical tasks grouped under 14 topics related to home objects or activities. Each topic contains three or four tasks, ranked by increasing difficulty. Unlike most home-based programmes, the physical therapists do not prescribe exercises, but help and coach the participant to build their own exercise programme while ensuring safety and security. The participants choose the tasks they want to perform, perform them once as a “test”, and evaluate the perceived difficulty on a five-level scale. Tasks that are evaluated as "very difficult" or "too difficult" are not included in their programme. The training focuses on: (i) encouragement of autonomy of the participant; (ii) the significance of evaluation of the perceived difficulty; (iii) coaching by the physical therapist; (iv) stimulation for adherence to exercises; not too many exercises at one session, but regularly; (v) the safety of the environment. Participants received a manual, including photographs and task descriptions, a set of cards representing each exercise with difficulty evaluation grids, and a digital tablet containing the programme application.

#### Reference programme

The Otago exercise programme is an individualised, partially supervised, home-based balance and strength training programme delivered by a trained physical therapist [14]. This programme has shown significant results in reducing the risk of death and falling in older community-dwelling adults aged ≥ 80 years [15]. This programme has also been shown to reduce the rate of secondary falls in people who have already fallen [16]. The programme contains 22 exercises with two to four levels of difficulty: five warm-up exercises, five exercises for muscle strengthening of the lower limbs, and 12 exercises for balance training. Physical therapists propose and adapt the level of the exercises over time. Participants received the manual, including photographs and descriptions of all exercises and cuff weights for strength training exercises.

#### Control programme

The Going Safely exercise programme is a self-administered home-based balance and strength training designed by a Swiss health insurance company [17]. It contains a booklet with safety advice and 12 exercise cards, comprising five exercises to be performed in a sitting position, six exercises to be performed in a standing position, and one stand-up exercise. Participants received the booklet at a single physical therapy session.

### Outcome measures

#### Primary outcome

The primary outcome was the incidence rate ratio of self-reported falls over the individual duration in the study. Falls were prospectively self-reported (number, date, circumstance, and severity) in monthly diaries. In addition, fall events (number, circumstance, and severity) were asked during the intermediate and final assessments. Falls were defined as “an unexpected event in which the participant comes to rest on the ground, floor, or lower level, with or without injury” [18]. The local coordinators contacted participants if they did not return their monthly diaries. They also recorded in the database description of falls and adverse events.

#### Secondary outcome

Secondary outcomes included fear of falling, basic functional mobility and balance, quality of life, and exercise adherence. Fear of falling was measured with the Falls Efficacy Scale-International [19] (range 16–64 points; a higher score indicating more concern about falling; 16–19 indicating low concern about falling, 20–27 indicating moderate concern, and 28–64 indicating high concern). Basic functional mobility and balance were assessed with the Short Physical Performance Battery (SPPB) [20] (including the Five Time Sit to Stand Test [20]) (range, 0–12; higher score indicating better performance; scores ≤ 10 predicting higher risk of mobility disability) [21], with the Timed Up and Go (TUG) test [22] (in seconds; lower scores indicating better performance; scores ≥ 12 s indicating risk of future falls) [23] and with Functional Reach (FR) test [24] (in cm; a higher score indicating better performance; score < 18.5 cm indicating fall risk) [25]. Quality of life was measured with the Older People’s Quality of Life Questionnaire-35 (OPQOL-35) [26, 27] (range 35–175; a higher score indicates better quality of life). Exercise adherence was prospectively assessed in monthly training diaries, in which date, duration, type of exercises and intensity of effort were noted. Exercise adherence was calculated as (time completed/total time expected) × 100. Total times expected for exercise were calculated as 360 min per month × 12 months.

### Data analysis

The incidence fall rate ratio was modelled with a negative binomial regression (primary analysis) [28]. For a significance level set at 5% and power of 80%, an assumed fall rate of 0.5 for the experimental group, 0.79 for the reference group, and 0.92 for the control group, a non-inferiority margin of 5%, and an assumed dropout rate of 15%, 162 participants were required in the reference and experimental groups, and 81 participants in the control group; a total of 405 participants [8]. The allocation scheme of 2:2:1 is based on ethical and statistical considerations, following the recommendations of Mütze et al. [29].

As secondary analyses, the incidence rate (falls per person-years) and its between-group differences were estimated with a negative binomial regression. The hazard ratios (Cox regression) were calculated for the first and second falls. Cumulative hazards for all falls and the cumulative incidence for the first and second falls were plotted. The negative binomial and Cox regression were calculated using Stata software version 17.0 (Stata Inc., College Station, TX, USA). Cumulative incidence plots were performed with R 4.2.2 (Foundation for Statistical Computing, Vienna, Austria. https://www.R-project.org/) and the packages survival [30] and ggsurvfit [31].

Secondary outcomes were analysed with linear regression. The between-group differences in percentage exercise adherence of the participants were compared using a Kruskal–Wallis test.

**Fig. 1:**
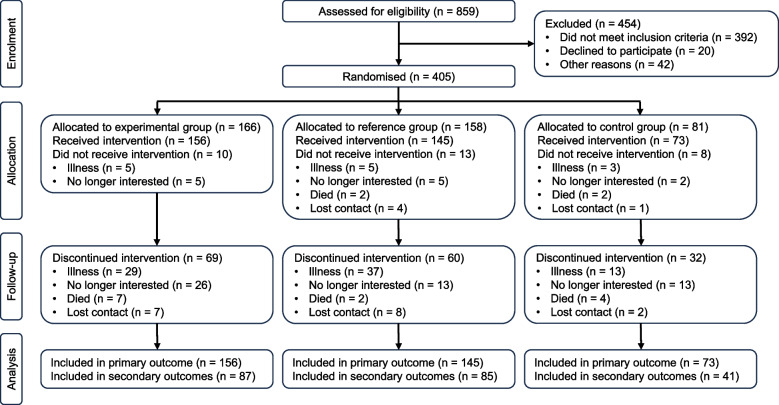
Flow of participants through the trial

All analyses were performed following an available data intention-to-treat analysis, from the initial (T_0_) to final assessment (T_2_). All participants were analysed in the group in which they were randomised, irrespective of whether they stopped the intervention early. Thirty-one participants who had only the baseline evaluation, but did not start the intervention, were excluded from the analysis (see Fig. 1 for reasons). All regressions were adjusted for the baseline value of the given outcome and the stratification variables rural/urban region, age group and risk of falls categories for adjustment.

## Results

Between August 2016 and November 2020, 859 potential participants were screened. Of these, 392 did not meet inclusion criteria, 20 declined to participate and 42 did not participate due to other reasons. A total of 405 participants were randomised into three groups: experimental group (*n* = 166), reference group (*n* = 158) and control group (*n* = 81). The flow of participants through the trial is shown in Fig. 1. A total of 374 participants were included in the primary analyses and 213 in secondary analyses. The reasons for drop-out are shown in Fig. 1.

Median follow-up time was 343 (IQR 170–361) days for the experimental group, 342 (IQR 205–358) days for the reference group, and 341 (IQR 152–364) days for the control group (Table 1). The final assessment of the last participant was in November 2021.

**Table 1:** Primary outcomes and secondary analyses

**Outcomes**	**Total (*n* = 374)**^**a**^	**Experimental group (*n* = 156)**	**Reference group (*n* = 145)**	**Control group (*n* = 73)**
Total exposure, d^b^				
Mean (SD)	271 (115)	271 (117)	279 (108)	258 (124)
Median (interquartile range)	342 (177–360)	343 (170–361)	342 (205–358)	341 (152–364)
No. of falls observed	382	141	199	42
**Primary Outcome**^**c**^				
Incidence rate ratio (95% CI), adjusted^d^		0.74 (0.49 to 1.12)	Comparator	0.43 (0.25 to 0.75)
Incidence rate ratio (95% CI), adjusted^d^		1.71 (0.98 to 2.99)	2.30 (1.33 to 4.00)	Comparator
Incidence rate ratio (95% CI), unadjusted		0.64 (0.42 to 0.97)	Comparator	0.41 (0.23 to 0.71)
Incidence rate ratio (95% CI), unadjusted		1.58 (0.90 to 2.77)	2.46 (1.41 to 4.29)	Comparator
**Secondary Analyses**				
Estimated falls per person-years (95% CI), (adjusted)	1.41 (1.14 to 1.67)	1.33 (0.94 to 1.73)	1.80 (1.27 to 2.32)	0.78 (0.41 to 1.15)
Estimated falls per person-years (95% CI), (unadjusted)	1.52 (1.23 to 1.81)	1.33 (0.93 to 1.72)	2.07 (1.46 to 2.67)	0.84 (0.44 to 1.24)
Incidence rate difference (95% CI), adjusted^e^		–0.46 (–1.12 to 0.19)	Comparator	–0.55 (–1.10 to –0.12)
Incidence rate difference (95% CI), adjusted^f^		0.55 (0.12 to 1.10)	1.02 (0.38 to 1.65)	Comparator
No. of falls, No. (%) of participants				
0	217 (58)	90 (58)	81 (56)	46 (63)
1	73 (20)	31 (20)	25 (17)	17 (23)
2	41 (11)	19 (12)	15 (10)	7 (10)
3	20 (5)	10 (6)	9 (6)	1 (1)
≥ 4	23 (6)	6 (4)	15 (10)	2 (3)
No. (%) of participants with ≥ 1 fall	157 (42)	66 (42)	64 (44)	27 (37)
No. of severe^g^ falls observed, n	59	26	24	9
Hazard ratio for repeated falls (95% CI)		0.68 (0.43 to 1.08)	Comparator	0.47 (0.29 to 0.75)
Hazard ratio for repeated falls (95% CI)		1.45 (0.91 to 2.31)	2.12 (1.33 to 3.40)	Comparator
Hazard ratio to first fall (95% CI)		0.92 (0.65 to 1.30)	Comparator	0.83 (0.53 to 1.31)
Hazard ratio to first fall (95% CI)		1.11 (0.70 to 1.74)	1.20 (0.76 to 1.89)	Comparator
Hazard ratio to second fall (95% CI)		0.81 (0.51 to 1.27)	Comparator	0.50 (0.25 to 1.01)
Hazard ratio to second fall (95% CI)		1.60 (0.79 to 3.25)	1.99 (0.99 to 4.00)	Comparator

^a^Thirty-one participants were omitted from these analyses because they did not start the intervention and never sent a fall diary and hence were never at risk of falling

^b^Values are without participants who had 0 follow-up time, i.e. without those who never received an intervention and never sent a falls diary (i.e. they were never at risk of falling)

^c^The differences in incidence rate ratio were 0.55 (95% CI 0.12 to 1.10; *P* = 0.045) for experimental group and 1.02 (95% CI 0.38 to 1.65; *P* = 0.002) for reference group versus control group; and 0.46 (95% CI –1.12 to 0.19; *P* = 0.166) for experimental group versus reference group

^d^Incidence rate ratio from adjusted negative binomial regression. Adjusted for the stratification variables: risk category (moderate or high), urban or rural region, age greater or lower than 80 years

^e^Incidence rate difference of the experimental group and control group versus the reference group

^f^Incidence rate difference of the experimental group and reference group versus the control group

^g^Severe falls means falls requiring medical treatment (i.e. with serious or moderate injuries)

All registered outcome measures are reported here. However, because the control group had the lowest fall incidence rate, the method deviated from the protocol by comparing the three groups pairwise instead of following Mütze [32].

### Characteristics of the participants

The mean age of participants was 79 (SD 7) years and 74% were women. Sixty-six percent of the whole sample fell in the 12 months preceding the start of the study and 48% were identified at high risk of falling. Table 2 presents the participants' characteristics.

**Table 2:** Sample characteristics

**Characteristics**	**Total (*n* = 405)**	**Experimental group (*n* = 166)**	**Reference group (*n* = 158)**	**Control group (*n* = 81)**
Age, years, mean (SD)	79 (7.0)	79 (7.0)	79 (6.6)	80 (7.6)
Sex, n (%)				
Male	106 (26)	47 (28)	41 (26)	18 (22)
Female	299 (74)	119 (72)	117 (74)	63 (78)
Body mass index, mean (SD)^a^	26 (4.8)	26 (4.8)	25 (4.9)	25 (4.6)
Living situation, n (%)				
Alone	216 (53)	82 (49)	88 (56)	46 (57)
Couple	158 (39)	74 (45)	54 (34)	30 (37)
With family members other than spouse	13 (3.2)	4 (2.4)	6 (3.8)	3 (3.7)
Sheltered apartment	3 (0.74)	2 (1.2)	1 (0.63)	0 (0.00)
Urban or rural, n (%)				
Rural	318 (79)	129 (78)	125 (79)	64 (79)
Urban	87 (21)	37 (22)	33 (21)	17 (21)
Self-reported neurological disease, n (%)	60 (15)	27 (16)	21 (13)	12 (15)
Self-reported musculoskeletal disease, n (%)	335 (83)	146 (88)	125 (79)	64 (79)
Self-reported urinary incontinence, n (%)	124 (31)	60 (36)	40 (25)	24 (30)
Self-reported vision impairment, n (%)	323 (80)	132 (80)	129 (82)	62 (77)
Mini-Mental State Examination, mean (SD)^b^	28 (1.7)	28 (1.7)	28 (1.7)	28 (1.7)
Number of medications, mean (SD)	3.9 (3.2)	4.3 (3.4)	3.6 (2.9)	3.6 (3.1)
Walking aids, n (%)	196 (48)	80 (48)	77 (49)	39 (48)
Fear of falling, n (%)				
Never	81 (20)	33 (20)	29 (18)	19 (23)
Sometimes	281 (69)	120 (72)	112 (71)	49 (60)
Always	43 (11)	13 (7.8)	17 (11)	13 (16)
Fallen in the last past 12 months, n (%)				
No	138 (34)	60 (36)	49 (31)	29 (36)
Yes	267 (66)	106 (64)	109 (69)	52 (64)
Number of falls in past 12 months, mean (SD)	1.4 (2.2)	1.3 (1.5)	1.6 (3.1)	1.2 (1.2)
Injurious falls in past 12 months, n (%)				
No injury	68 (17)	27 (16)	26 (16)	15 (19)
Light injury (< 3 days)	53 (13)	19 (11)	20 (13)	14 (17)
Moderate injury (medical consultation)	50 (12)	19 (11)	24 (15)	7 (8.6)
Severe injury (emergency consultation or hospital)	96 (24)	41 (25)	39 (25)	16 (20)
Fall Risk Category (STEADI CDC)^c^, n (%)				
Low	64 (16)	23 (14)	29 (18)	12 (15)
Moderate	146 (36)	63 (38)	51 (32)	32 (40)
High	195 (48)	80 (48)	78 (49)	37 (46)

^a^Weight in kilograms divided by height in meters squared

^b^Mini-Mental State Examination score range from 0 (worst) to 30 (best); scores of 24–30 are considered unimpaired

^c^STEADI CDC indicates Stopping Elderly Accidents, Deaths & Injuries from Centers for Disease Control and Prevention

### Primary outcome and secondary analyses

During a mean follow-up of 271 (SD 115) days, 141 falls occurred among 66 participants in the experimental group, 199 falls among 64 participants in the reference group, and 42 falls among 27 participants in the control group. A total of 157 participants fell at least once (66 in the experimental group; 64 in the reference group; 27 in the control group) and 36 severe falls occurred in the experimental group, 24 in the reference group, and 9 in the control group (Table 1). No adverse events related to the intervention were reported.

The adjusted fall incidence rate ratio was 0.74 (95% CI 0.49 to 1.12) in the experimental group and 0.43 (95% CI 0.25 to 0.75) in the control group, compared with the reference group. The adjusted incidence rate differences were –0.46 (95% CI –1.12 to 0.19; *P* = 0.166) for the experimental group versus the reference group; and 0.55 (95% CI 0.12 to 1.10; *P* = 0.045) for the experimental group and 1.02 (95% CI 0.38 to 1.65; *P* = 0.002) for the reference group versus the control group. The adjusted estimated incidence rate was 1.33 (95% CI 0.94 to 1.73) per person-years in the experimental group, 1.80 (95% CI 1.27 to 2.32) per person-years in the reference group, and 0.78 (95% CI 0.41 to 1.15) per person-years in the control group.

**Fig. 2:**
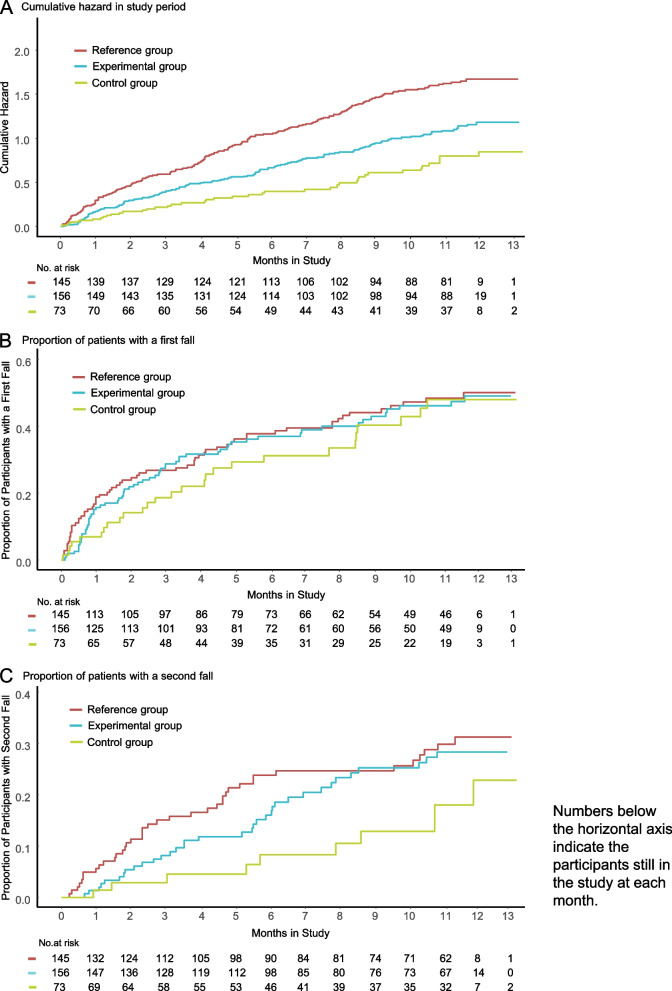
Cumulative hazard for repeated falls and cumulative incidence of first and second fall

The fall incidence rate was lower in the control group compared with the 2 partially supervised groups (Fig. 2A), with a statistically significant incidence rate difference between the reference group and the control group (1.02 (95% CI 0.38 to 1.65; *P* = 0.0027)) but no significant difference between the experimental group and the control group (0.55 (95% CI 0.12 to 1.19, *P* = 0.045)).

Regarding the hazard ratio for the first fall, there were no significant differences between the 3 groups (Fig. 2B) with a hazard ratio of 0.92 (95% CI 0.65 to 1.30; *P* = 0.647) for the experimental group versus the reference group, 1.11 (95% CI 0.70 to 1.74; *P* = 0.658) for the experimental group versus the control group, and 1.20 (95% CI 0.76 to 1.89; *P* = 0.428) for the reference group versus the control group.

The hazard ratios for the second fall were not statistically significant, with a hazard ratio of 0.81 (95% CI 0.51 to 1.27; *P* = 0.355) for the experimental group versus the reference group, 0.50 (95% CI 0.25 to 1.01; *P* = 0.053) for the control group versus the reference group, and 1.60 (95% CI 0.79 to 3.25; *P* = 0.190) for the experimental group versus the control group.

For repeated falls, the fall rate was the lowest in the control group and comparable between the experimental and reference groups. The hazard ratio for repeated falls showed a significant difference only for the control group versus the reference group, with a hazard ratio of 0.47 (95% CI 0.29 to 0.75; *P* = 0.002). For the experimental group versus the reference group, the hazard ratio was 0.68 (95% CI 0.43 to 1.08; *P* = 0.103) and 1.45 (95% CI 0.91 to 2.31; *P* = 0.123) for the experimental group versus the control groups (Fig. 2C).

### Secondary outcomes

The SPPB scores improved significantly in the experimental group (mean difference between the groups of 0.45 (95% CI 0.05 to 0.86; *P* = 0.027) points) and the reference group (mean difference between the groups: 0.46 (95% CI 0.06 to 0.86; *P* = 0.024) points) compared with the control group (Table 3). Likewise, the Five Time Sit to Stand Test scores improved significantly in the experimental group (mean difference between the groups: –2.15 (95% CI –3.48 to 0.82; *P* = 0.002) seconds) and reference group (mean difference between the groups: –2.11 (95% CI –3.44 to –0.78; *P* = 0.002) seconds) compared with the control group (Table 3). Significant within-group differences were observed for fear of falling in the reference group (*P* < 0.001), for SPPS in the experimental group (*P* = 0.024) and for the reference group (*P* < 0.001), and for FTSTS in the experimental and reference groups (*P* < 0.001) (eTable 1 in Additional file 1).

**Table 3:** Between-group differences in secondary outcomes from baseline to 12 months

**Outcomes**	**Experimental group versus reference group**		**Experimental group versus control group**		**Reference group versus control group**	
	^**a**^**Mean difference (95% CI)**	***P* value**	^**a**^**Mean difference (95% CI)**	***P* value**	^**a**^**Mean difference (95% CI)**	***P* value**
Falls Efficacy Scale-International score^b^ (*n* = 213)	0.13 (–1.34 to 1.6)	0.862	–1.27 (–3.12 to 0.58)	0.179	–1.4 (–3.25 to 0.46)	0.139
Short Physical Performance Battery score^c^ (*n* = 213)	–0.01 (–0.33 to 0.31)	0.963	0.45 (0.05 to 0.86)	0.027^*^	0.46 (0.06 to 0.86)	0.024^*^
Five Time Sit to Stand Test, s^d^ (*n* = 213)	–0.04 (–1.11 to 1.03)	0.941	–2.15 (–3.48 to –0.82)	0.002^*^	–2.11 (–3.44 to –0.78)	0.002^*^
Functional Reach test score, cm^e^ (*n* = 209)	0.78 (–1.73 to 3.29)	0.539	0.57 (–2.65 to 3.79)	0.728	–0.21 (–3.45 to 3.02)	0.896
Timed Up and Go Test score^f^, s (*n* = 213)	–0.38 (–1.34 to 0.59)	0.443	–0.61 (–1.83 to 0.6)	0.321	–0.24 (–1.45 to 0.98)	0.701
Older People's Quality of Life Questionnaire-35 score^g^ (*n* = 212)	1.68 (–1.47 to 4.83)	0.295	3.9 (–0.06 to 7.86)	0.053	2.23 (–1.73 to 6.18)	0.269

*n* = number of participants

^*^ Bold numbers: experimental and reference group were statistically significantly better than control group with *P* < 0.05

^a^Adjusted mean differences; adjusted for baseline values of the dependent variable and risk category, age category, rural or urban

^b^Falls Efficacy Scale-International: higher values in the score indicate more concerns; a positive difference in the table indicates that improvement was better in the right-hand intervention; a negative difference indicates that improvement was better in the left-hand intervention (here from left to right favouring reference group, experimental group, and reference group, respectively)

^c^Short Physical Performance Battery: higher values in the score indicate better performance; a positive difference indicates that improvement was better in the left-hand intervention; a negative difference indicates that improvement was better in the right-hand intervention (here from left to right favouring reference group, experimental group, and reference group, respectively)

^d^Five Time Sit to Stand Test: lower score indicates better performance; a negative difference indicates that improvement was better in the right-hand intervention (here from left to right favouring experimental group, experimental group, and reference group, respectively)

^e^Functional Reach Test score: higher score indicates better performance; a positive difference indicates that improvement was better in the left-hand intervention; a negative difference indicates that improvement was better in the right-hand intervention (here from left to right favouring experimental group, experimental group, and control group, respectively)

^f^Timed Up and Go Test score: lower score indicates better performance; a positive difference indicates that improvement was better in the left-hand intervention (here from left to right favouring experimental group, experimental group, and reference group, respectively)

^g^Older People's Quality of Life Questionnaire-35 score: higher score indicates better quality of life; a positive difference indicates that improvement was better in the left-hand intervention; a negative difference indicates that improvement was better in the right-hand intervention (here from left to right favouring experimental group, experimental group, and reference group, respectively)

In terms of quality of life, there were no statistically significant differences between the three groups (eTable 1 in Additional file 1). Median adherence to exercise was 58.2% in the experimental group, 73.7% in the reference group, and 59.7% in the control group, with no significant differences between any groups (eFigure 1 in Additional file 1).

## Discussion

This randomised controlled trial including 405 older adults at risk for falls found that partially supervised exercise programmes were not more effective than a self-administered programme in reducing the incidence of falls in identified at-risk older adults. In contrast to most studies that compare an intervention with usual care (i.e. no specific intervention for fall prevention), the control group received a self-administered exercise programme [33].

The primary hypothesis of this study, that partially supervised programmes are better at preventing falls than a self-administered programme, could not be confirmed. Furthermore, the experimental programme showed no significant improvement compared with the reference programme in reducing the incidence rate of falls. The confidence interval included a predefined non-inferiority margin of 5%. Therefore, we cannot confirm the second hypothesis, which states that the experimental group is not less effective than the reference group. The reference programme showed the highest incidence rate of falls, although in a previous randomised controlled trial, this programme demonstrated efficacy compared with a control group with no specific intervention in secondary fall prevention [16]. This could be explained by the probability that not all falls were reported by participants in the control group, and/or the fact that 59% of dropouts in the control group were high-risk fallers, compared with 46% in the intervention group and 49% in the reference group (see eTable 2, eTable 3, and eFigure 2 in Additional file 1).

This study showed significant improvement in secondary outcomes, consistent with the results of a previous systematic review [4] and the randomised controlled trial of Sherrington et al. [34]. Lacroix et al. also observed an improvement in physical parameters in participants who have been supervised compared with participants who trained unsupervised [35]. However, the current results support the conclusion that the experimental programme is not less effective than the well-established reference programme regarding improvement in physical functioning of participants.

This study has several limitations. First, there was a high rate of dropouts or withdrawals (47%) between randomisation and month 12. This could be partially explained by disruption caused by the emergence of the COVID-19 (SARS-CoV-2) pandemic during the trial. Secondly, the control group showed an unexpectedly high dropout rate in participants with a high risk of falling before month 1. This affected the originally planned randomisation and the results. Thirdly, 79% of participants were from rural areas, and hence the results may not represent urban areas. Fourthly, follow-up of the participants in the control group did not include as many in-person meetings as those in the intervention and reference groups. Another limitation was the self-reporting of the falls. It is possible that the self-reporting was higher in the intervention group than in the control group like reported in Mackenzie and al. [36]. Furthermore, the protocol planned to simultaneously demonstrate superiority of the intervention to reference programme and the reference to control programme.

Given the lower falls incidence rate of the control group compared to both the intervention and reference groups, we had to deviated from the planned analysis.

Finally, the sample size calculation might have been based on overly optimistic assumptions regarding the between group differences in the falls incidence rates. There is also evidence that trials seeking to demonstrate reductions in fall rates in the community require minimum 500 participants [37].

In conclusion, the self-administered home-based exercise programme for older adults resulted in the lowest fall incidence rate, but also the highest drop-out rate of participants at high risk of falling. Both partially supervised exercise programmes resulted in statistically significant improvements in physical performance compared with the self-administered exercise programme. These findings support the use of partially supervised home-based exercise programmes in older adults at risk of falling to improve physical function, but not to reduce falls.

### Supplementary Information

**Additional file 1. **eAddenda containing eTables and eFigures.

**Additional file 2. **CONSORT checklist.

## Data Availability

The datasets used and/or analysed during the current study are available from the corresponding author on reasonable request.
