# Supplementary material for: Partially supervised exercise programmes for fall prevention improve physical performance of older people at risk of falling: a three-armed multi-centre randomised controlled trial
Source: BMC Geriatr. 2024 Apr 3;24:311. doi: 10.1186/s12877-024-04927-0 (PMC10993430; doi:10.1186/s12877-024-04927-0)
Supplement: Supplementary file 1 — Additional file 1. eAddenda containing eTables and eFigures. [file 12877_2024_4927_MOESM1_ESM.docx]

**eAddenda**

List of eTables and eFigures

**eTable 1.** Within-group differences in secondary outcomes from baseline to Month 12.

**eTable 2.** Fall risk categories in the three groups at baseline, Month 0, and Month 12.

**eTable 3.** Dropouts in fall risk categories in the three groups between randomisation and baseline, and between baseline and Month 12.

**eFigure1.** Rate of adherence to exercise recommendations.

**eFigure 2.** Changes in risk category in the three groups during the study.

**eTable 1.** Within-group differences in secondary outcomes from baseline to Month 12.

|  | **Experimental Group (randomized n=87)** | | | | **Reference Group (randomized n=85)** | | | | **Control Group (randomized n=41)** | | | |
| --- | --- | --- | --- | --- | --- | --- | --- | --- | --- | --- | --- | --- |
| Outcomes | Baseline | Month 12 | Within-group difference | *P* value | Baseline | Month 12 | Within-group difference | *P* value | Baseline | Month 12 | Within-group difference | *P* value |
| Falls Efficacy Scale-International score, mean (SD) | 26.6 (7.4) | 24.1 (5.9) | –2.4 (6.1) | 0.139 | 26.7 (7.7) | 24.1 (5.7) | –2.6 (5.4) | **<0.001**** | 26.9 (10.4) | 25.4 (11) | –1.5 (5.8) | 0.117 |
| Number of participants | n = 87 | | | | n = 85 | | | | n = 41 | | | |
| Short Physical Performance Battery score, mean (SD) | 7.9 (1.4) | 8.4 (1.3) | 0.5 (1.4) | **0.024*** | 7.9 (1.3) | 8.4 (1.3) | 0.5 (1.2) | **<0.001**** | 8.1 (1.3) | 8.1 (1.1) | 0 (1.2) | 0.789 |
| Number of participants | n = 87 | | | | n = 85 | | | | n = 41 | | | |
| Five Time Sit to Stand, mean (SD), s | 16.0 (4.9) | 13.8 (3.3) | 2.2 (4.4) | **<0.001**** | 15.7 (5.8) | 13.7 (4.8) | 2.0 (4.1) | **<0.001**** | 14.8 (15.3) | 15.6 (6.2) | –0.5 (3.3) | 0.409 |
| Number of participants^a^ | n = 78 (83) | | | | n = 72 (76) | | | | n = 37 (38) | | | |
| Functional Reach test score, mean (SD), cm | 25.9 (9.2) | 26.1 (8.2) | 0.2 (9.8) | 0.896 | 27 (12.1) | 25.4 (9.3) | –1.5 (13.9) | 0.320 | 24.7 (9.3) | 25.6 (9.8) | 0.9 (11.1) | 0.619 |
| Number of participants | n =87 | | | | n = 84 | | | | n = 38 | | | |
| Time Up and Go Test score, mean (SD), s | 11.9 (4.1) | 11.3 (3.8) | –0.6 (2.8) | 0.701 | 12.1 (5.1) | 11.9 (5.7) | –0.2 (4.3) | 0.590 | 12.4 (5.1) | 12,2 (5) | –0.2 (2.7) | 0.632 |
| Number of participants | n = 87 | | | | n = 85 | | | | n = 41 | | | |
| Older People's Quality of Life Questionnaire-35 score, mean (SD) | 141.9 (15.1) | 145.3 (14.7) | 3.4 (10.7) | 0.296 | 141.7 (15.3) | 143.3 (14.9) | 1.7 (10.3) | 0.140 | 140.3 (18.9) | 140.1 (19.8) | –0.2 (13.4) | 0.917 |
| Number of participants | n=86 | | | | n=85 | | | | n=41 | | | |

*Statistically significant with *P* < .005.

**Statistically significant with *P* < .001.

Bold numbers : statistically significant

SD = standard deviation; n = number of participants who had evaluation at baseline and at 12 months; s = seconds; cm = centimeters.

^a^Experimental group : 5 additional participants were able to perform the test at month 12 (n = 83). When included in the analysis, the mean is 14.0 (3.3) at 12 months. Reference group: 4 additional participants, mean 13.8 (4.8). Control group: 1 additional participant, mean 15.6 (6.2).

**eTable 2.** Fall risk categories in the 3 groups at baseline (randomized), Month 0, and Month 12.

| **A** | **Percentage and number of participants in each fall risk category, all participants** | | | | | | | | | | | |
| --- | --- | --- | --- | --- | --- | --- | --- | --- | --- | --- | --- | --- |
|  |  | **Experimental group** | | |  | **Reference group** | | |  | **Control group** | | |
|  | Risk category | Randomized | Month 0 | Month 12 |  | Randomized | Month 0 | Month 12 |  | Randomized | Month 0 | Month 12 |
|  | Low-risk, % (n) | 14 (23) | 13 (20) | 24 (21) |  | 18 (29) | 19 (28) | 36 (31) |  | 15 (12) | 16 (12) | 37 (15) |
|  | Moderate risk, % (n) | 38 (63) | 38 (59) | 67 (58) |  | 32 (51) | 32 (46) | 48 (41) |  | 40 (32) | 41 (30) | 41 (17) |
|  | High-risk, % (n) | 48 (80) | 49 (77) | **9 (8)** |  | 49 (78) | 49 (71) | **15 (13)** |  | 46 (37) | 42 (31) | **22 (9)** |
|  | Total, % (n) | 100 (166) | 100 (156) | 100 (87) |  | 100 (158) | 100 (145) | 100 (85) |  | 100 (81) | 100 (73) | 100 (41) |
|  |  |  |  |  |  |  |  |  |  |  |  |  |
| **B** | **Percentage and number of participants in each fall risk category, Month 0 included only the participants who performed the final evaluation** | | | | | | | | | | | |
|  |  |  | **Experimental group** | |  |  | **Reference group** | |  |  | **Control group** | |
|  | Risk category |  | Month 0 | Month 12 |  |  | Month 0 | Month 12 |  |  | Month 0 | Month 12 |
|  | Low-risk, % (n) |  | 11 (10) | 24 (21) |  |  | 20 (17) | 36 (31) |  |  | 22 (9) | 37 (15) |
|  | Moderate risk, % (n) |  | 39 (34) | **67 (58)** |  |  | 33 (28) | **48 (41)** |  |  | 41 (17) | **41 (17)** |
|  | High-risk, % (n) |  | 49 (43) | 9 (8) |  |  | 47 (40) | 15 (13) |  |  | 37 (15) | 22 (9) |
|  | Total, % (n) |  | 100 (87) | 100 (87) |  |  | 100 (85) | 100 (85) |  |  | 100 (41) | 100 (41) |

% = percentage of participants; n = number of participants.

Bold numbers: At month 12 the percentage of participants at high risk of falling is lowest in the experimental group (9%) compared to the reference group (15%) and the control group (22%) and the percentage of participants at moderate risk of falling is highest in the experimental group (67%) compared to the reference group (48%) and the control group (41%).

**eTable 3.** Dropouts in fall risk categories in the 3 groups between randomization and baseline, and between baseline and Month 12.

|  |  | **Experimental group** | | |  | **Reference group** | | |  | | **Control group** | | |
| --- | --- | --- | --- | --- | --- | --- | --- | --- | --- | --- | --- | --- | --- |
| Risk category | | Dropouts^a^ | Dropouts^b^ | Dropouts^c^ |  | Dropouts^a^ | Dropouts^b^ | Dropouts^c^ | |  | Dropouts^a^ | Dropouts^b^ | Dropouts^c^ |
| Low-risk, % (n) | | 13 (3) | 50 (10) | **57 (13)** |  | 3 (1) | 39 (11) | **41 (12)** | |  | 0 (0) | 25 (3) | **25 (3)** |
| Moderate risk, % (n) | | 6 (4) | 50 (25) | 46 (29) |  | 10 (5) | 39 (18) | 45 (23) | |  | 6 (2) | 43 (13) | 47 (15) |
| High-risk, % (n) | | 4 (3) | 44 (34) | **46 (37)** |  | 9 (7) | 44 (31) | **49 (38)** | |  | 16 (6) | 52 (16) | **59 (22)** |
| Total, % (n) | | 6 (10) | 44 (69) | 48 (79) |  | 8 (13) | 41 (60) | 46 (73) | |  | 10 (8) | 44 (32) | 49 (40) |

% = percentage of participants; n = number of participants.

The percentages in the columns Dropouts ^a,b,c^ can be interpreted as risk to dropout for each fall risk category.

Bold numbers: The risk of dropout is not evenly distributed across the 3 fall risk categories. The experimental group has the highest percentage of dropout among participants with low fall risk (57%) compared to the reference group (41%) and the control group (25%). Conversely, the experimental group has the lowest percentage of dropout among participants at high fall risk of falling (46%) compared to the reference group (49%) and the control group (59%).

^a^Dropouts between randomization and Month 0 in percentages based on randomized participants.

^b^Dropouts between Month 0 and Month 12 in percentages based on participants who started the study.

^c^Dropouts between randomization and Month 12 in percentage based on randomized participants.

**eFigure1.** Rate of Adherence to exercise recommendations.

The lower and upper boundaries of the boxes represent the 25th and 75th percentiles. The horizontal black line indicates the median. The black diamond indicates the mean. The whiskers cover 1.5 times the interquartile range above and below the box (i.e., distance in the unit of measurement between the 25th and 75th percentiles. The points indicate participants outside 1.5 times interquartile range above and below the box. The notches indicate a confidence interval around the median (+/– 1.58 times the interquartile range divided by the square root of n). One outlier at 476% adherence was cut from the image. P = p value; Ø = Mean; p50 = Median.

**eFigure 2.** Changes in risk category in the 3 groups during the study
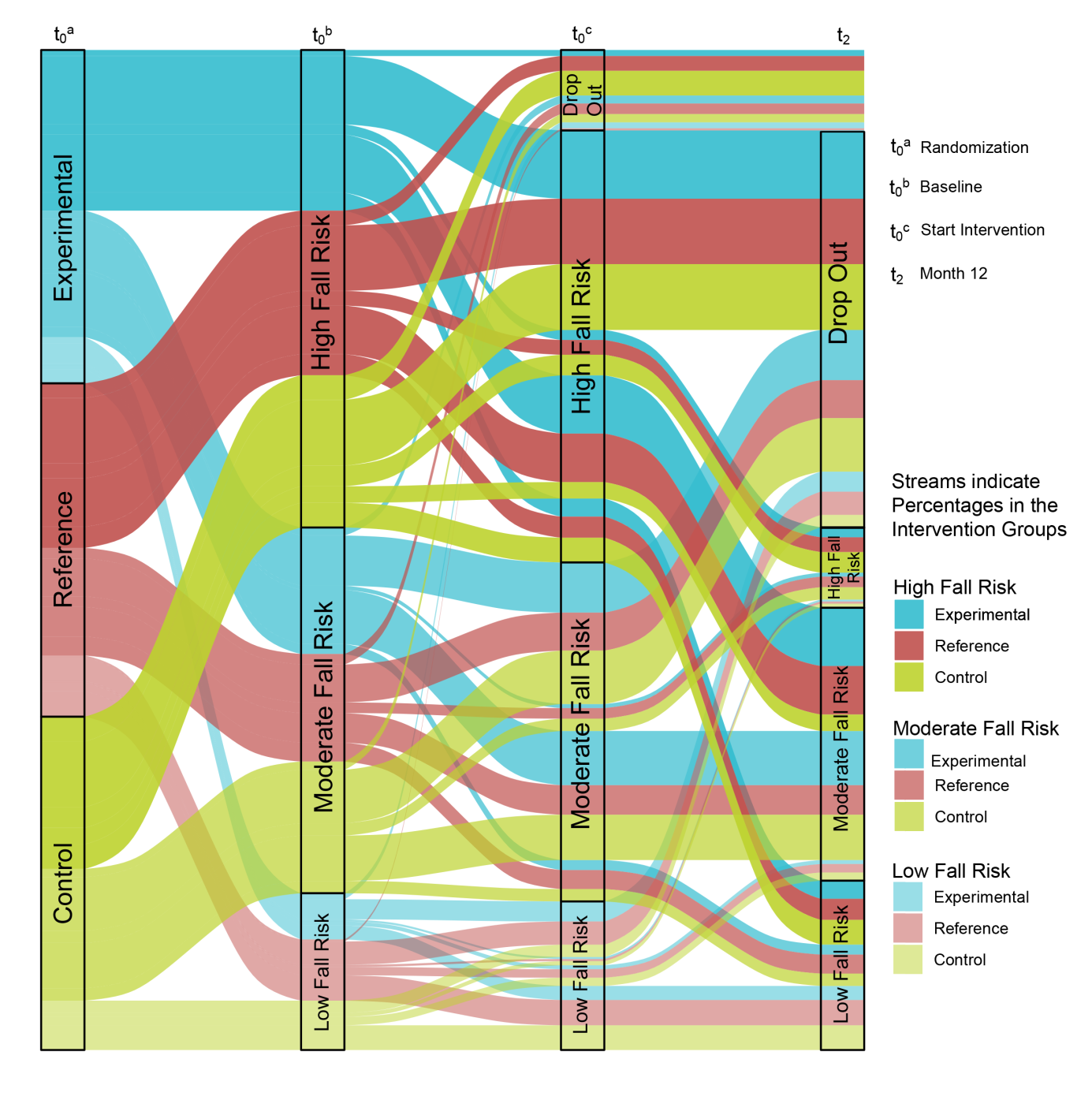
.
