# Supplementary material for: Partially supervised exercise programmes for fall prevention improve physical performance of older people at risk of falling: a three-armed multi-centre randomised controlled trial
Source: BMC Geriatr. 2024 Apr 3;24:311. doi: 10.1186/s12877-024-04927-0 (PMC10993430; doi:10.1186/s12877-024-04927-0)
Supplement: Supplementary file 2 — Additional file 2. CONSORT checklist. [file 12877_2024_4927_MOESM2_ESM.docx]

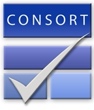
Checklist for Reporting of Multi-Arm Parallel-Group Randomized Trials: Extension of the CONSORT 2010 Statement^*^

| Section/Topic | Item No | Checklist item | Reported on page No |
| --- | --- | --- | --- |
| Title and abstract | | | |
|  | 1a | Identification as a multi-arm randomised trial in the title or an indication of the number of treatment groups that the participants were randomly assigned to | 1 |
|  | 1b | Structured summary of trial design, methods, results, and conclusions (for specific guidance see CONSORT for abstracts) / Specification of the number of treatment groups; details of any groups added or dropped | 2 |
| Introduction | | | |
| Background and objectives | 2a | Scientific background and explanation of rationale / Rational for using a multi-arm design | 4 |
|  | 2b | Specific objectives or hypotheses / Specification of the research question referring to all of the treatment groups – Clear statement of all hypotheses to be tested and the primary comparisons involved | 4 |
| Methods | | | |
| Trial design | 3a | Description of trial design (such as parallel, factorial) including allocation ratio / Specification of the number of treatment groups | 5 |
|  | 3b | Important changes to methods after trial commencement (such as eligibility criteria), with reasons | N/A |
| Participants | 4a | Eligibility criteria for participants | 6 |
|  | 4b | Settings and locations where the data were collected | 6 |
| Interventions | 5 | The interventions for each group with sufficient details to allow replication, including how and when they were actually administered | 7 |
| Outcomes | 6a | Completely defined pre-specified primary and secondary outcome measures, including how and when they were assessed | 8 |
|  | 6b | Any changes to trial outcomes after the trial commenced, with reasons | N/A |
| Sample size | 7a | How sample size was determined / Planned sample size with details of how it was determined for each primary comparison | 9 |
|  | 7b | When applicable, explanation of any interim analyses and stopping guidelines | N/A |
| Randomisation: |  |  |  |
| Sequence generation | 8a | Method used to generate the random allocation sequence | 5 |
|  | 8b | Type of randomisation; details of any restriction (such as blocking and block size) | 5 |
| Allocation concealment mechanism | 9 | Mechanism used to implement the random allocation sequence (such as sequentially numbered containers), describing any steps taken to conceal the sequence until interventions were assigned | 5 |
| Implementation | 10 | Who generated the random allocation sequence, who enrolled participants, and who assigned participants to interventions | 5 |
| Blinding | 11a | If done, who was blinded after assignment to interventions (for example, participants, care providers, those assessing outcomes) and how | 5 |
|  | 11b | If relevant, description of the similarity of interventions | N/A |
| Statistical methods | 12a | Statistical methods used to compare groups for primary and secondary outcomes / Explicity state if no adjustments for multiplicity were applied; if adjustments were applied, stat the method used | 9-10 |
|  | 12b | Methods for additional analyses, such as subgroup analyses and adjusted analyses | 9-10 |
| Results | | | |
| Participant flow (a diagram is strongly recommended) | 13a | For each group, the numbers of participants who were randomly assigned, received intended treatment, and were analysed for the primary outcome | Figure 1 |
|  | 13b | For each group, losses and exclusions after randomisation, together with reasons | Figure 1 |
| Recruitment | 14a | Dates defining the periods of recruitment and follow-up / If periods of recruitment and follow-up are different across treatment groups (e, groups were added or dropped), the periods of recruitment and follow-up, reason(s) for the differences, and any statistical implications should be described | 5; 10 |
|  | 14b | Why the trial ended or was stopped | N/A |
| Baseline data | 15 | A table showing baseline demographic and clinical characteristics for each group | Table 2 |
| Numbers analysed | 16 | For each group, number of participants (denominator) included in each analysis and whether the analysis was by original assigned groups | In all tables |
| Outcomes and estimation | 17a | For each primary and secondary outcome, results for each group, and the estimated effect size and its precision (such as 95% confidence interval) / Results for each prespecified comparison of treatment groups | 10-13 |
|  | 17b | For binary outcomes, presentation of both absolute and relative effect sizes is recommended | Table 1 |
| Ancillary analyses | 18 | Results of any other analyses performed, including subgroup analyses and adjusted analyses, distinguishing pre-specified from exploratory | Table 1 |
| Harms | 19 | All important harms or unintended effects in each group (for specific guidance see CONSORT for harms) | 10 |
| Discussion | | | |
| Limitations | 20 | Trial limitations, addressing sources of potential bias, imprecision, and, if relevant, multiplicity of analyses | 14 |
| Generalisability | 21 | Generalisability (external validity, applicability) of the trial findings | 14 |
| Interpretation | 22 | Interpretation consistent with results, balancing benefits and harms, and considering other relevant evidence | 14 |
| Other information | | |  |
| Registration | 23 | Registration number and name of trial registry | 2 |
| Protocol | 24 | Where the full trial protocol can be accessed, if available | https://doi.org/10.1186/s12877-018-1021-y |
| Funding | 25 | Sources of funding and other support (such as supply of drugs), role of funders | 15 |

Citation: Juszczak, E., Altman, D. G., Hopewell, S., & Schulz, K. (2019). Reporting of multi-arm parallel-group randomized trials: extension of the CONSORT 2010 statement. Jama, 321(16), 1610-1620.

*We strongly recommend reading this statement in conjunction with the CONSORT 2010 Explanation and Elaboration for important clarifications on all the items. If relevant, we also recommend reading CONSORT extensions for cluster randomised trials, non-inferiority and equivalence trials, non-pharmacological treatments, herbal interventions, and pragmatic trials. Additional extensions are forthcoming: for those and for up to date references relevant to this checklist, see [www.consort-statement.org](http://www.consort-statement.org).
